# Supplementary material for: Tay-Sachs and Sandhoff Diseases: Diffusion tensor imaging and correlational fiber tractography findings differentiate late-onset GM2 Gangliosidosis
Source: medRxiv. 2024 Dec 16:2024.12.13.24318793. Preprint. [Version 1] doi: 10.1101/2024.12.13.24318793 (PMC11722463; doi:10.1101/2024.12.13.24318793)
Supplement: Supplement 1 [file NIHPP2024.12.13.24318793v1-supplement-1.pdf]

medRxiv preprint doi: https://doi.org/10.1101/2024.12.13.24318793; this version posted December 16, 2024. The copyright holder for this preprint (which was not certified by peer review) is the author/funder, who has granted medRxiv a license to display the preprint in perpetuity.
This article is a US Government work. It is not subject to copyright under 17 USC 105 and is also made available for use under a CC0 license.

Tay-Sachs and Sandhoff Diseases: Diffusion tensor imaging and correlational fiber tractography findings differentiate late-onset GM2 Gangliosidosis Supplementary Material

Table of Contents

Methods……………………………………………………………………………….…*20*

Supplement A: Natural History Study Participant Characteristics……………..………..………*20*

Results……………………………………………………………………………………...*21*

Supplement B: Diffusion Tensor Imaging Analysis……………………………………………...*21*

Supplement C: Diffusion Tensor Imaging Analysis……………………………………………...*30*

Supplement D: Correlational Fiber Tractography….…………………………………………….…*32*

medRxiv preprint doi: https://doi.org/10.1101/2024.12.13.24318793; this version posted December 16, 2024. The copyright holder for this preprint (which was not certified by peer review) is the author/funder, who has granted medRxiv a license to display the preprint in perpetuity. This article is a US Government work. It is not subject to copyright under 17 USC 105 and is also made available for use under a CC0 license.

Supplementary Methods

Supplement A: Natural History Study Participant Characteristics

Table A1. Natural History Study DWI Cohort (n = 16), specific ages redacted per MedArXiv requirements

Participant	GM2 Sub-type	Baseline Age (years old)	Scan #2 Age (years old)	Scan #3 Age (years old)	Scan #4 Age (years old)	Average DWI interval (years)	Number of DWI Scans
NHS 32	LOTS	26-30	26-30	31-35	N/A	2	3
NHS 46	LOTS	41-45	41-45	41-45	N/A	1	3
NHS 47	LOTS	51-55	51-55	56-60	N/A	2.5	3
NHS 50	LOTS	26-30	26-30	N/A	N/A	2	2
NHS 103	LOTS	41-45	41-45	N/A	N/A	2	2
NHS 56	LOTS	26-30	26-30	26-30	31-35	1.67	4
NHS 70	LOTS	41-45	41-45	N/A	N/A	2	2
NHS 65	LOTS	31-35	36-40	N/A	N/A	2	2
NHS 57	LOTS	56-60	61-65	N/A	N/A	6	2
NHS 75	LOSD	51-55	56-60	N/A	N/A	5	2
NHS 68	LOSD	41-45	46-50	N/A	N/A	5	2
NHS 67	LOTS	26-30	31-35	N/A	N/A	2	2
NHS 55	LOTS	26-30	26-30	26-30	31-35	1.67	4
NHS 43	LOSD	61-65	61-65	N/A	N/A	1	2
NHS 45	LOSD	46-50	51-55	51-55	51-55	1.67	4
NHS 104	LOTS	66-70	N/A	N/A	N/A	N/A	1

medRxiv preprint doi: https://doi.org/10.1101/2024.12.13.24318793; this version posted December 16, 2024. The copyright holder for this preprint (which was not certified by peer review) is the author/funder, who has granted medRxiv a license to display the preprint in perpetuity. This article is a US Government work. It is not subject to copyright under 17 USC 105 and is also made available for use under a CC0 license.

Supplementary Results

Supplement B: Diffusion Tensor Imaging Analysis Supplement Figures

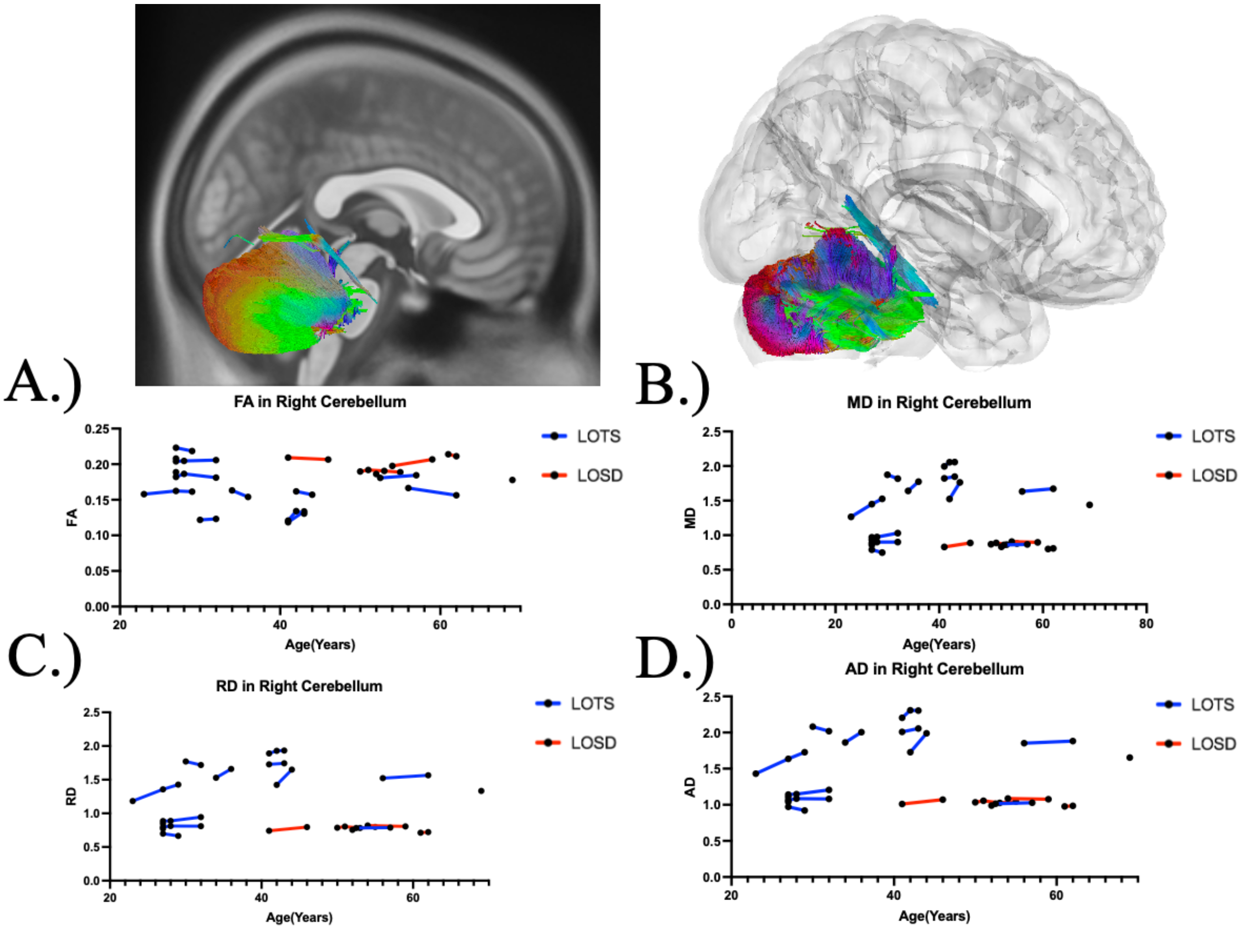

Figure B1. Atlas based fiber tractography of the right cerebellum demonstrating age related effects on A.) fractional anisotropy B.) mean diffusivity between C.) radial diffusivity D.) axial diffusivity between Tay-Sachs patients (blue) and Sandhoff patients (red). Tay-Sachs patients demonstrated decreased FA ($\chi^2(1) = 5.50$, $p = 0.019$) and increased MD ($\chi^2(1) = 8.56$, $p = 0.0034$), RD ($\chi^2(1) = 8.55$, $p = 0.0035$), and AD ($\chi^2(1) = 8.57$, $p = 0.0034$) compared to Sandhoff patients in fiber tracts in the right cerebellum when age was accounted for.

medRxiv preprint doi: https://doi.org/10.1101/2024.12.13.24318793; this version posted December 16, 2024. The copyright holder for this preprint (which was not certified by peer review) is the author/funder, who has granted medRxiv a license to display the preprint in perpetuity. This article is a US Government work. It is not subject to copyright under 17 USC 105 and is also made available for use under a CC0 license.

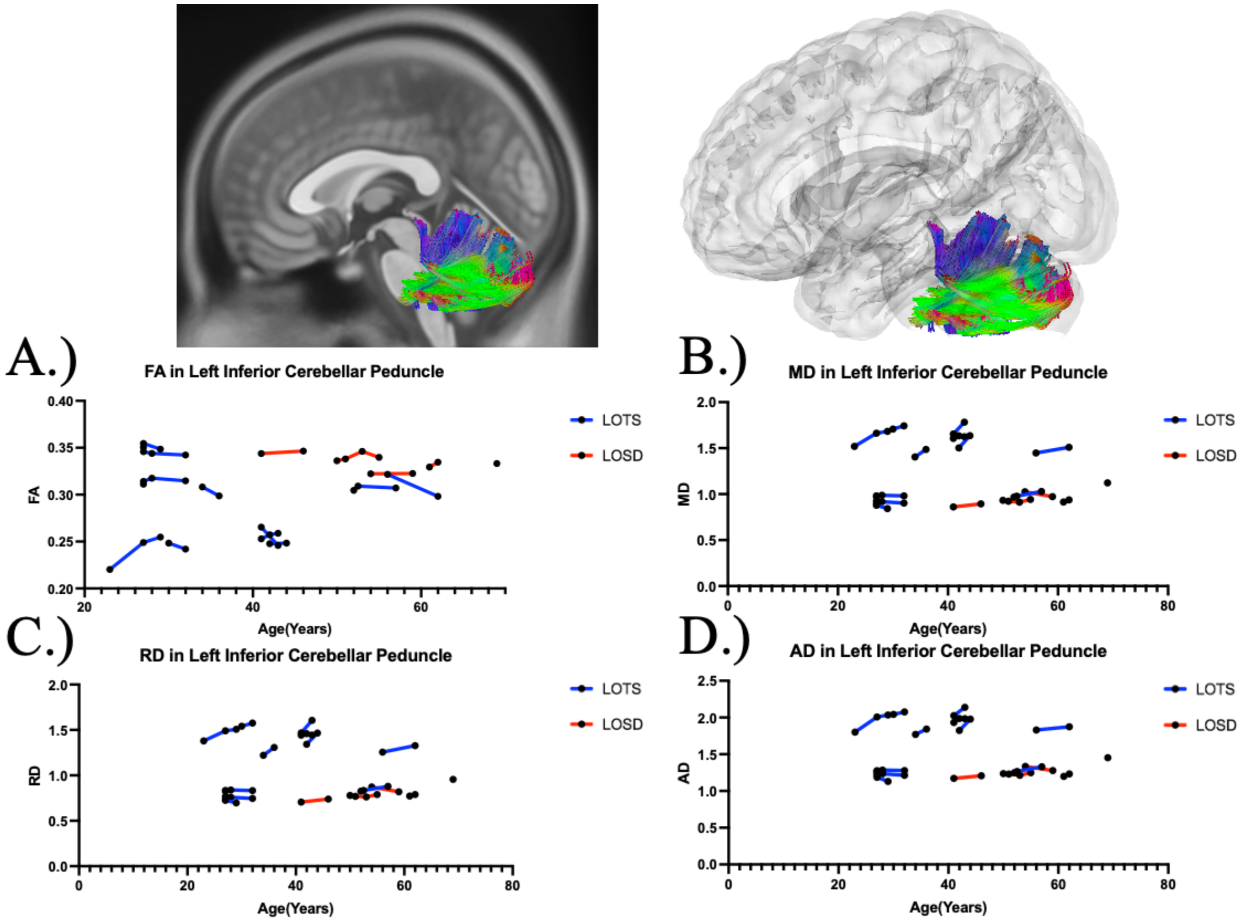

Fig B2. Atlas based fiber tractography of the left inferior cerebellar peduncle demonstrating age related effects on A.) fractional anisotropy B.) mean diffusivity between C.) radial diffusivity D.) axial diffusivity between Tay-Sachs patients (blue) and Sandhoff patients (red). Tay-Sachs patients demonstrated no difference in FA ($\chi^2(1) = 3.10$, $p = 0.078$) and increased MD ($\chi^2(1) = 7.02$, $p = 0.0081$), RD ($\chi^2(1) = 6.85$, $p = 0.0089$), and AD ($\chi^2(1) = 7.26$, $p = 0.0070$) compared to Sandhoff patients in fiber tracts in the left inferior cerebellar peduncle when age was accounted for.

medRxiv preprint doi: https://doi.org/10.1101/2024.12.13.24318793; this version posted December 16, 2024. The copyright holder for this preprint (which was not certified by peer review) is the author/funder, who has granted medRxiv a license to display the preprint in perpetuity. This article is a US Government work. It is not subject to copyright under 17 USC 105 and is also made available for use under a CC0 license.

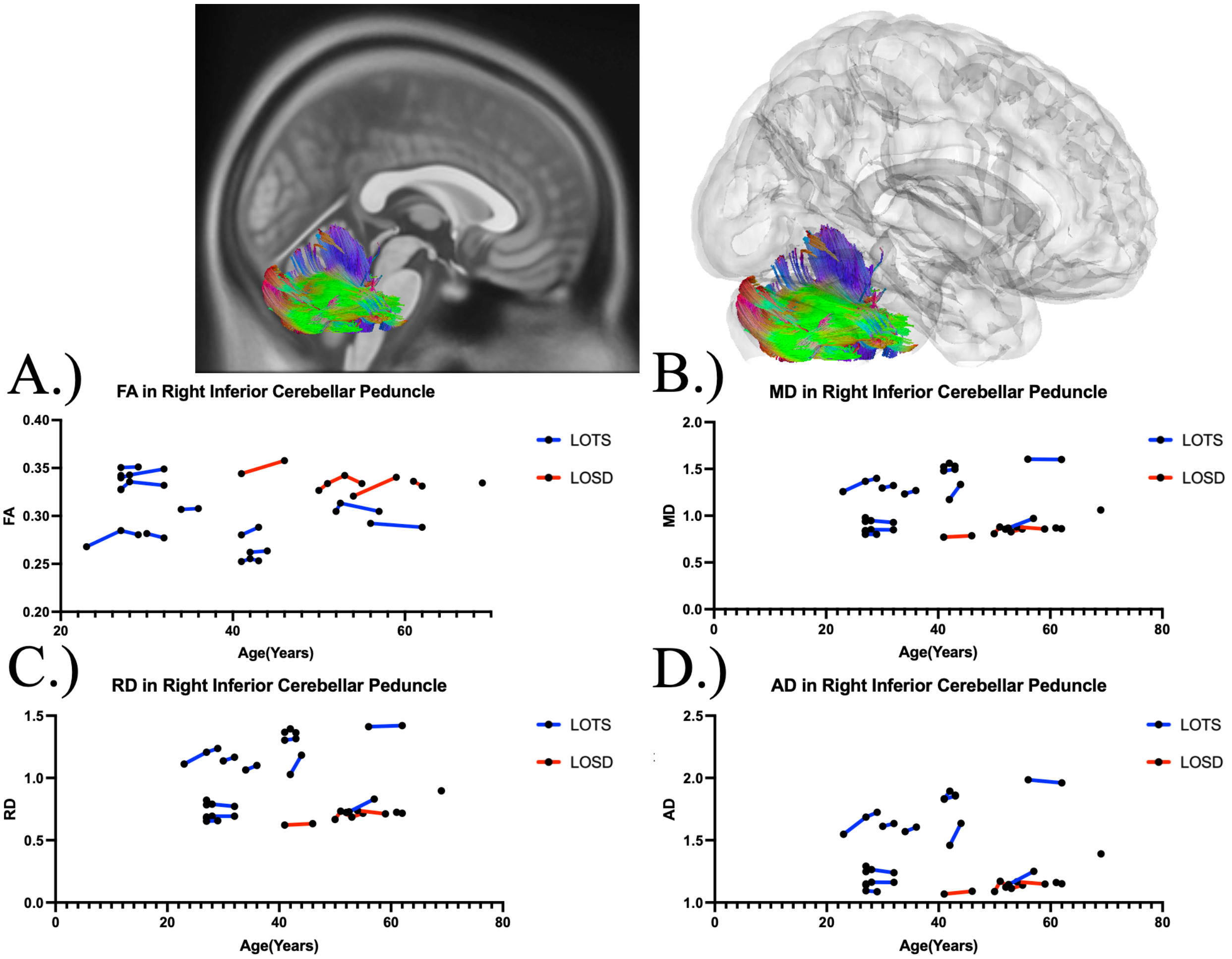

Fig B3. Atlas Based Fiber Tractography of the right inferior cerebellar peduncle demonstrating age related effects on A.) fractional anisotropy B.) mean diffusivity between Tay-Sachs patients (blue) and Sandhoff patients (red). Tay-Sachs patients demonstrated no difference in FA ($\chi^2(1) = 1.89$, $p = 0.17$) and increased MD ($\chi^2(1) = 8.55$, $p = 0.0035$), RD ($\chi^2(1) = 8.39$, $p = 0.0038$), and AD ($\chi^2(1) = 8.79$, $p = 0.0030$) compared to Sandhoff patients in fiber tracts in the right inferior cerebellar peduncle when age was accounted for.

medRxiv preprint doi: https://doi.org/10.1101/2024.12.13.24318793; this version posted December 16, 2024. The copyright holder for this preprint (which was not certified by peer review) is the author/funder, who has granted medRxiv a license to display the preprint in perpetuity.
This article is a US Government work. It is not subject to copyright under 17 USC 105 and is also made available for use under a CC0 license.

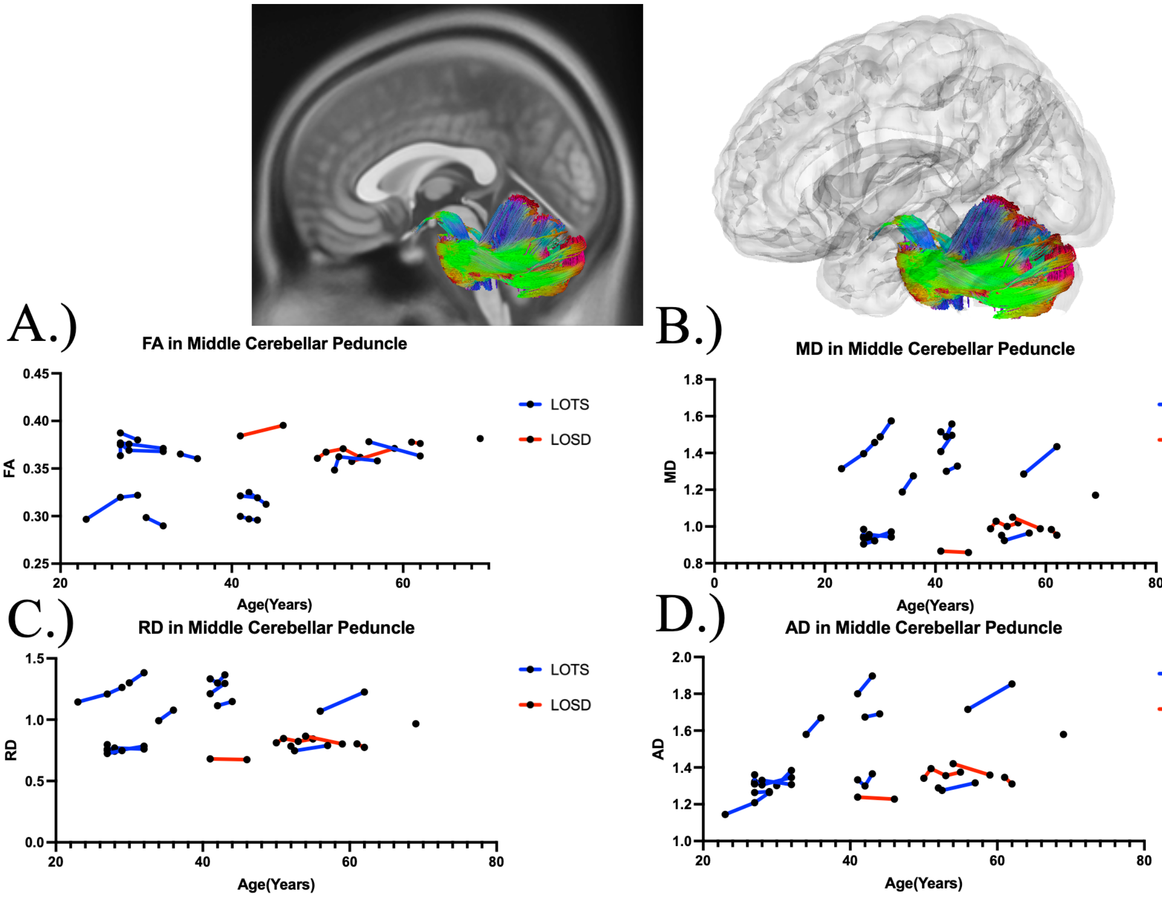

Fig B4. Atlas Based Fiber Tractography of the middle cerebellar peduncle demonstrating age related effects on A.) fractional anisotropy B.) mean diffusivity between Tay-Sachs patients (blue) and Sandhoff patients (red). Tay-Sachs patients demonstrated no difference in FA ($\chi^2(1) = 1.65$, $p = 0.20$) and increased MD ($\chi^2(1) = 6.81$, $p = 0.0091$), RD ($\chi^2(1) = 6.55$, $p = 0.010$), and AD ($\chi^2(1) = 7.22$, $p = 0.0072$) compared to Sandhoff patients in fiber tracts in the middle cerebellar peduncle when age was accounted for.

medRxiv preprint doi: https://doi.org/10.1101/2024.12.13.24318793; this version posted December 16, 2024. The copyright holder for this preprint (which was not certified by peer review) is the author/funder, who has granted medRxiv a license to display the preprint in perpetuity. This article is a US Government work. It is not subject to copyright under 17 USC 105 and is also made available for use under a CC0 license.

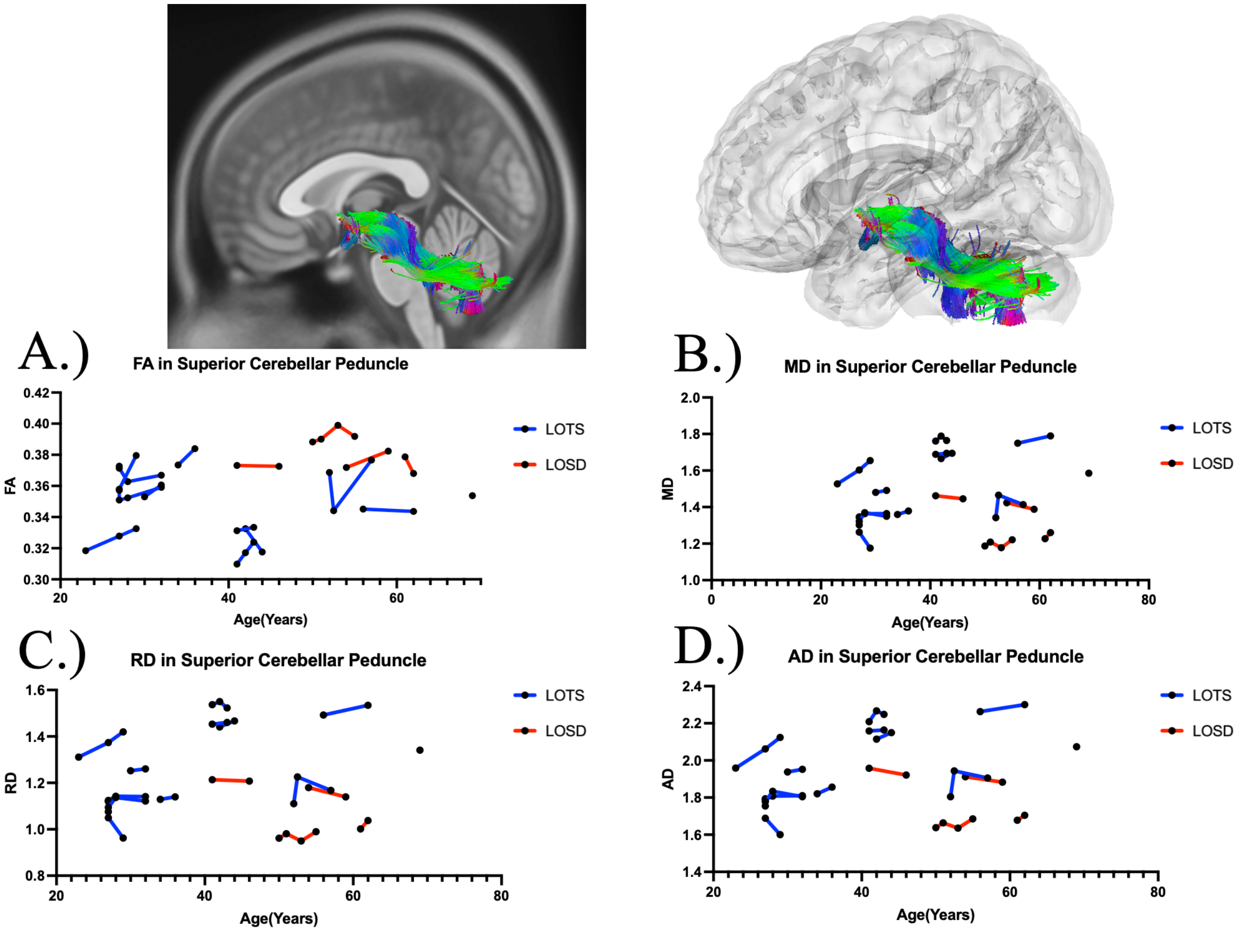

Fig B5. Atlas Based Fiber Tractography of the superior cerebellar peduncle demonstrating age related effects on A.) fractional anisotropy B.) mean diffusivity between Tay-Sachs patients (blue) and Sandhoff patients (red). Tay-Sachs patients demonstrated lower FA ($\chi^2(1) = 4.80$, $p = 0.028$) and increased MD ($\chi^2(1) = 7.19$, $p = 0.0073$), RD ($\chi^2(1) = 7.18$, $p = 0.0074$), and AD ($\chi^2(1) = 7.09$, $p = 0.0078$) compared to Sandhoff patients in fiber tracts in the superior cerebellar peduncle when age was accounted for.

medRxiv preprint doi: https://doi.org/10.1101/2024.12.13.24318793; this version posted December 16, 2024. The copyright holder for this preprint (which was not certified by peer review) is the author/funder, who has granted medRxiv a license to display the preprint in perpetuity. This article is a US Government work. It is not subject to copyright under 17 USC 105 and is also made available for use under a CC0 license.

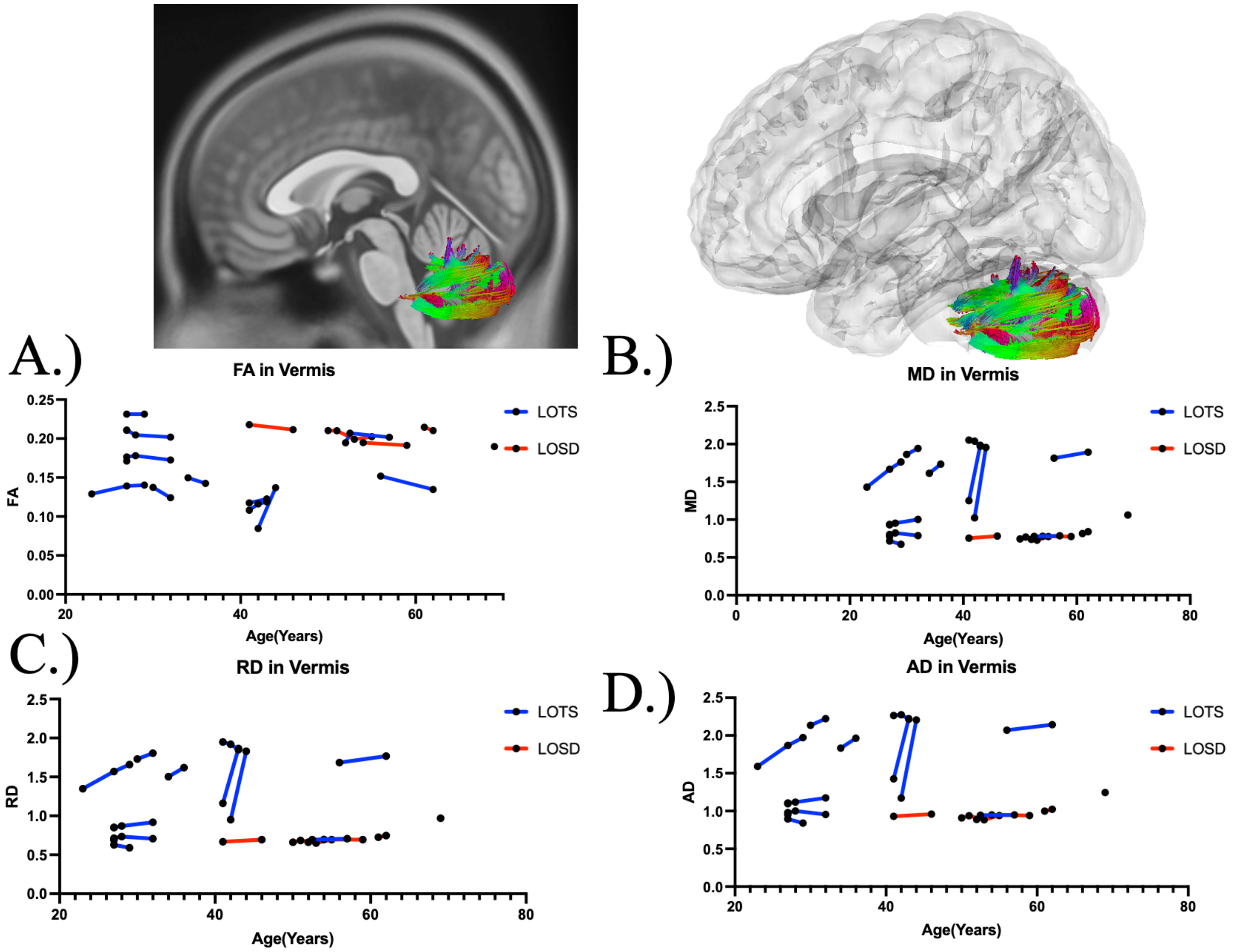

Fig B6. Atlas Based Fiber Tractography of the cerebellar vermis demonstrating age related effects on A.) fractional anisotropy B.) mean diffusivity between Tay-Sachs patients (blue) and Sandhoff patients (red). Tay-Sachs patients demonstrated lower FA ($\chi^2(1) = 5.05$, $p = 0.025$) and increased MD ($\chi^2(1) = 6.62$, $p = 0.010$), RD ($\chi^2(1) = 6.70$, $p = 0.0096$), and AD ($\chi^2(1) = 6.47$, $p = 0.011$) compared to Sandhoff patients in fiber tracts in the cerebellar vermis when age was accounted for.

medRxiv preprint doi: https://doi.org/10.1101/2024.12.13.24318793; this version posted December 16, 2024. The copyright holder for this preprint (which was not certified by peer review) is the author/funder, who has granted medRxiv a license to display the preprint in perpetuity.
This article is a US Government work. It is not subject to copyright under 17 USC 105 and is also made available for use under a CC0 license.

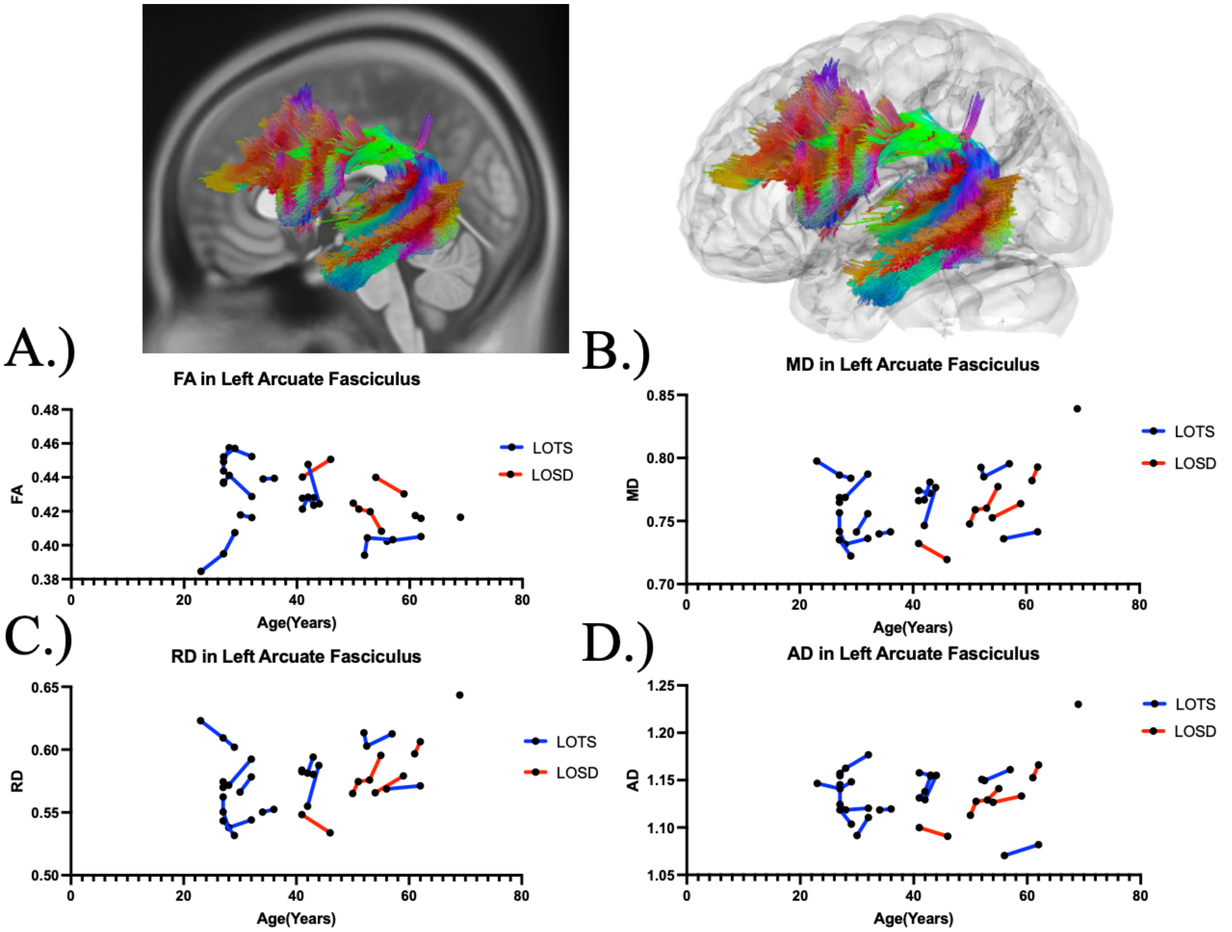

Fig B7. Atlas Based Fiber Tractography of the left arcuate fasciculus demonstrating age related effects on A.) fractional anisotropy B.) mean diffusivity between Tay-Sachs patients (blue) and Sandhoff patients (red). There was no statistical difference between Tay-Sachs and Sandhoff patients in FA ($\chi^2(1) = 0.92$, $p = 0.34$), MD ($\chi^2(1) = 2.65$, $p = 0.10$), RD ($\chi^2(1) = 2.66$, $p = 0.10$), or AD ($\chi^2(1) = 2.02$, $p = 0.16$) in fiber tracts in the left arcuate fasciculus.

medRxiv preprint doi: https://doi.org/10.1101/2024.12.13.24318793; this version posted December 16, 2024. The copyright holder for this preprint (which was not certified by peer review) is the author/funder, who has granted medRxiv a license to display the preprint in perpetuity.
This article is a US Government work. It is not subject to copyright under 17 USC 105 and is also made available for use under a CC0 license.

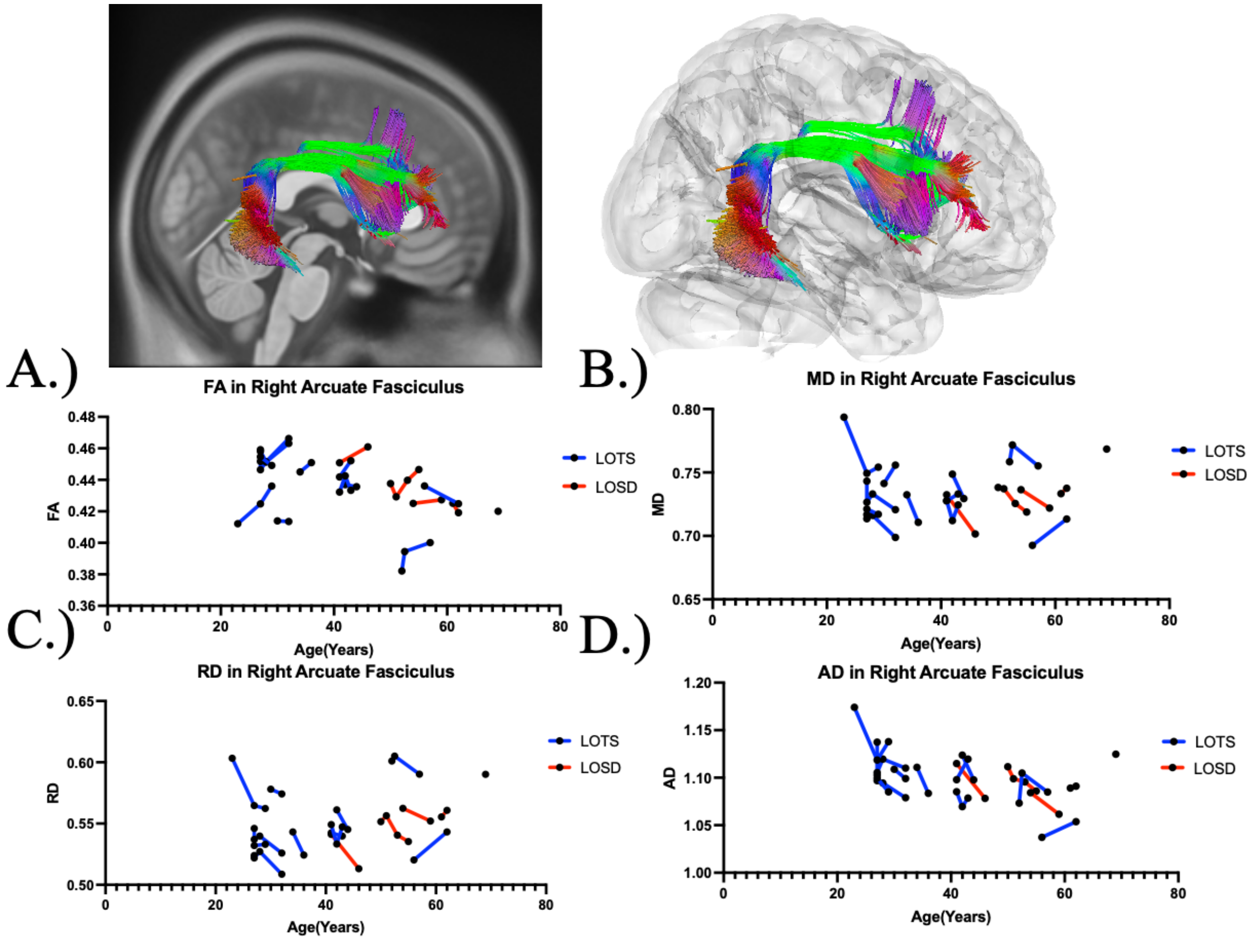

Fig B8. Atlas Based Fiber Tractography of the right arcuate fasciculus demonstrating age related effects on A.) fractional anisotropy B.) mean diffusivity between Tay-Sachs patients (blue) and Sandhoff patients (red). There was no statistical difference between Tay-Sachs and Sandhoff patients in FA ($\chi^2(1) = 0.044$, $p = 0.834$), MD ($\chi^2(1) = 0.12$, $p = 0.73$), RD ($\chi^2(1) = 0.14$, $p = 0.71$), or AD ($\chi^2(1) = 0.079$, $p = 0.78$) in fiber tracts in the right arcuate fasciculus.

medRxiv preprint doi: https://doi.org/10.1101/2024.12.13.24318793; this version posted December 16, 2024. The copyright holder for this preprint (which was not certified by peer review) is the author/funder, who has granted medRxiv a license to display the preprint in perpetuity.
This article is a US Government work. It is not subject to copyright under 17 USC 105 and is also made available for use under a CC0 license.

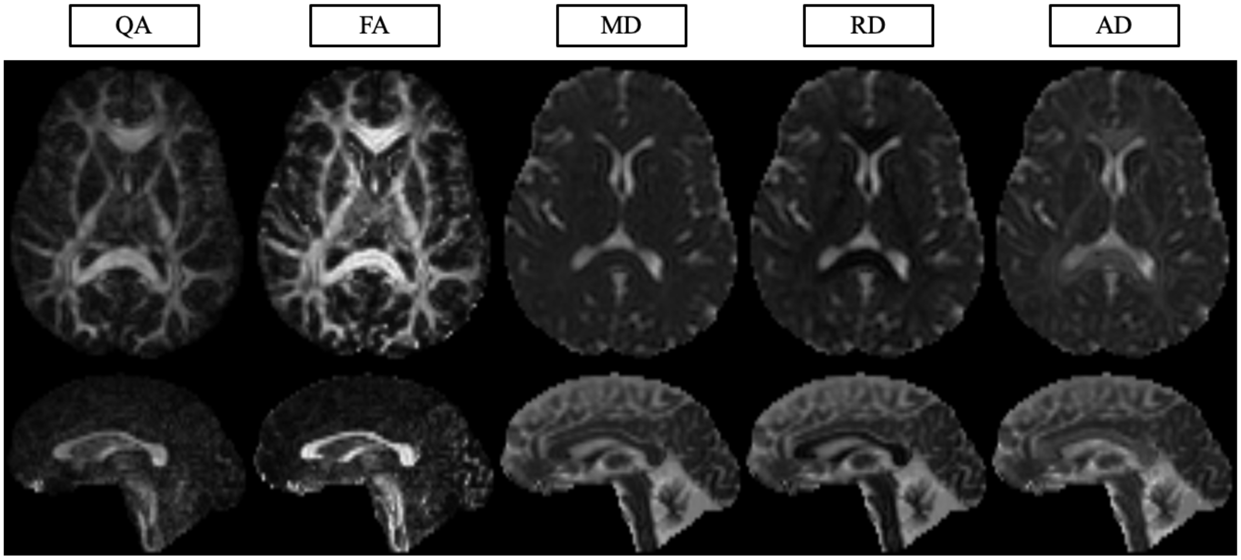

Figure B9. Quantitative Anisotropy (QA), Fractional Anisotropy (FA), Mean Diffusivity (MD), Radial Diffusivity (RD), and Axial Diffusivity (AD) imaging.

medRxiv preprint doi: https://doi.org/10.1101/2024.12.13.24318793; this version posted December 16, 2024. The copyright holder for this preprint (which was not certified by peer review) is the author/funder, who has granted medRxiv a license to display the preprint in perpetuity. This article is a US Government work. It is not subject to copyright under 17 USC 105 and is also made available for use under a CC0 license.

Supplement C. Diffusion Tensor Imaging Analysis of RD, AD, and QA

Table C1. Diffusion Tensor Imaging Results of RD in Atlas Fiber Tractography Pathways evaluating differences between Tay-Sachs and Sandhoff Patients.

Pathway	Estimate	Standard Error	$\chi^2(1)$	*p-value* ($>\chi^2$)
**Whole Brain**	-0.0943	0.0337	7.28	**0.0070**
**Left Cerebellum**	-0.7112	0.232	8.51	**0.0035**
**Right Cerebellum**	-0.6976	0.226	8.55	**0.0035**
**Left Inferior Cerebellar Peduncle**	-0.4781	0.178	6.85	**0.0089**
**Right Inferior Cerebellar Peduncle**	-0.4245	0.139	8.39	**0.0038**
**Middle Cerebellar Peduncle**	-0.3327	0.128	6.55	**0.010**
**Superior Cerebellar Peduncle**	-0.2590	0.0934	7.18	**0.0074**
**Vermis**	-0.6959	0.269	6.70	**0.0096**
**Corpus Callosum**	0.0028	0.0239	0.02	0.90
**Left Arcuate Fasciculus**	-0.0220	0.0142	2.66	0.10
**Right Arcuate Fasciculus**	-0.0023	0.0162	0.14	0.71

Table C2. Diffusion Tensor Imaging Results of AD in Atlas Fiber Tractography Pathways evaluating differences between Tay-Sachs and Sandhoff Patients.

Pathway	Estimate	Standard Error	$\chi^2(1)$	*p-value* ($>\chi^2$)
**Whole Brain**	-0.1003	0.0346	7.86	**0.0051**
**Left Cerebellum**	-0.7554	0.252	8.25	**0.0041**
**Right Cerebellum**	-0.7511	0.244	8.57	**0.0034**
**Left Inferior Cerebellar Peduncle**	-0.5458	0.196	7.26	**0.0070**
**Right Inferior Cerebellar Peduncle**	-0.4845	0.155	8.79	**0.0030**
**Middle Cerebellar Peduncle**	-0.3712	0.135	7.22	**0.0072**
**Superior Cerebellar Peduncle**	-0.2810	0.103	7.09	**0.0078**
**Vermis**	-0.7520	0.297	6.47	**0.011**
**Corpus Callosum**	0.0027	0.0286	0.01	0.91
**Left Arcuate Fasciculus**	-0.0268	0.0199	2.02	0.16
**Right Arcuate Fasciculus**	0.0039	0.0138	0.08	0.78

medRxiv preprint doi: https://doi.org/10.1101/2024.12.13.24318793; this version posted December 16, 2024. The copyright holder for this preprint (which was not certified by peer review) is the author/funder, who has granted medRxiv a license to display the preprint in perpetuity. This article is a US Government work. It is not subject to copyright under 17 USC 105 and is also made available for use under a CC0 license.

Table C3. Diffusion Tensor Imaging Results of QA in Atlas Fiber Tractography Pathways evaluating differences between Tay-Sachs and Sandhoff Patients.

Pathway	Estimate	Standard Error	$\chi^2(1)$	*p-value* ($>\chi^2$)
**Whole Brain**	-0.0084	0.0109	0.68	0.41
**Left Cerebellum**	0.0337	0.0185	3.57	0.06
**Right Cerebellum**	0.0193	0.0168	1.53	0.22
**Left Inferior Cerebellar Peduncle**	0.0208	0.0201	1.15	0.28
**Right Inferior Cerebellar Peduncle**	0.0024	0.0169	0.02	0.90
**Middle Cerebellar Peduncle**	0.0240	0.0206	1.47	0.22
**Superior Cerebellar Peduncle**	0.0065	0.0155	0.16	0.69
**Vermis**	0.0310	0.0191	2.89	0.09
**Corpus Callosum**	-0.0214	0.0137	2.65	0.10
**Left Arcuate Fasciculus**	-0.0107	0.0132	0.73	0.39
**Right Arcuate Fasciculus**	-0.0211	0.0143	2.39	0.12

medRxiv preprint doi: https://doi.org/10.1101/2024.12.13.24318793; this version posted December 16, 2024. The copyright holder for this preprint (which was not certified by peer review) is the author/funder, who has granted medRxiv a license to display the preprint in perpetuity. This article is a US Government work. It is not subject to copyright under 17 USC 105 and is also made available for use under a CC0 license.

Supplement D: Correlational Fiber Tractography Analysis, Supplement Figures

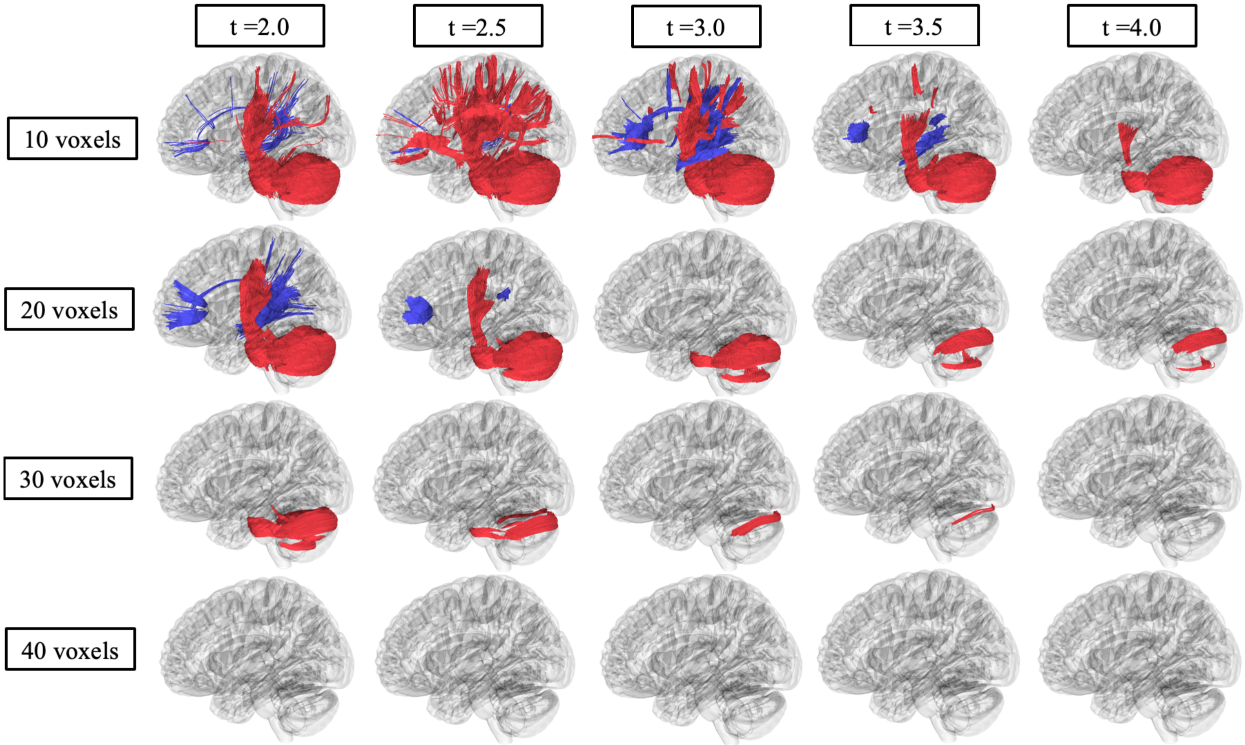

Figure D1. Correlational fiber tractography assessed differences in quantitative anisotropy (QA) in Sandhoff and Tay-Sachs patients at varying length (voxels) and T thresholds. Fiber tracts shown in red were evaluated to have a higher quantitative anisotropy in Sandhoff patients compared to Tay-Sachs patients and were observed primarily in the cerebellum (FDR<0.05). Fiber tracts shown in blue were evaluated to have a higher quantitative anisotropy in Tay-Sachs patients compared to Sandhoff patients.

medRxiv preprint doi: https://doi.org/10.1101/2024.12.13.24318793; this version posted December 16, 2024. The copyright holder for this preprint (which was not certified by peer review) is the author/funder, who has granted medRxiv a license to display the preprint in perpetuity. This article is a US Government work. It is not subject to copyright under 17 USC 105 and is also made available for use under a CC0 license.

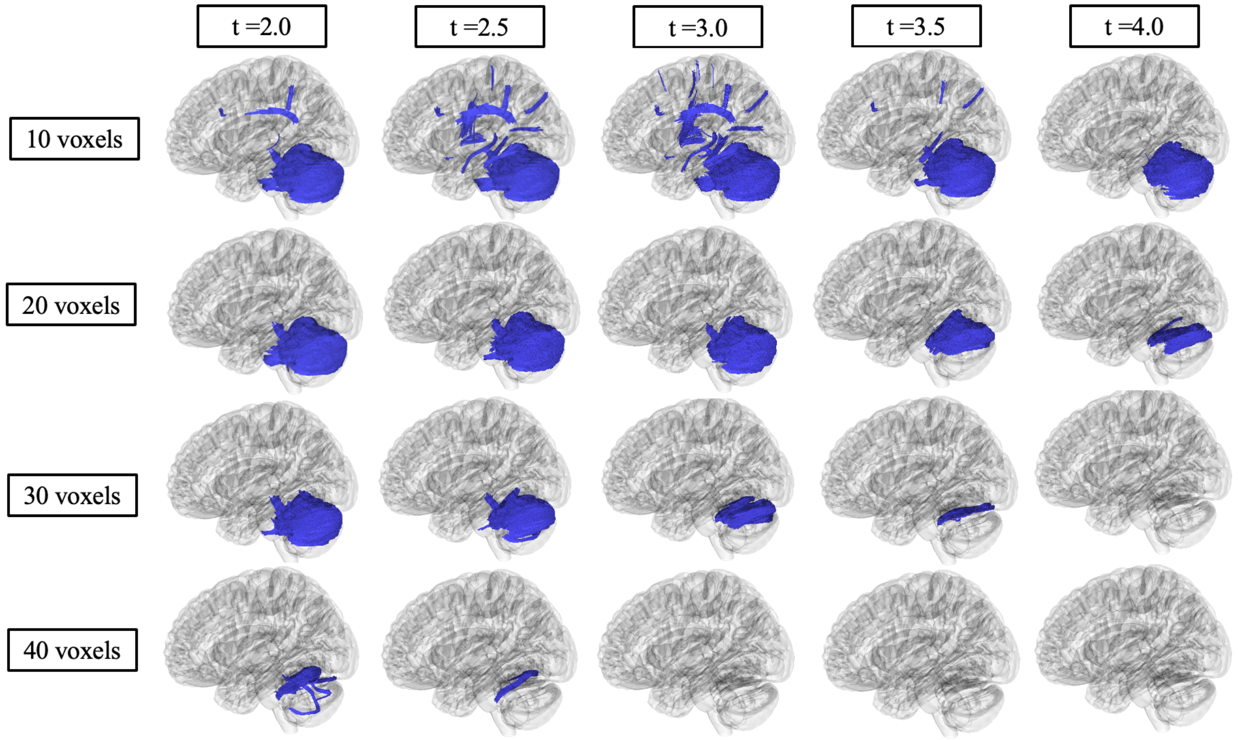

Figure D2. Correlational fiber tractography assessed differences in radial diffusivity (RD) in Sandhoff and Tay-Sachs patients at varying length (voxels) and T thresholds. Fiber tracts shown in blue were evaluated to have a higher radial diffusivity in Tay-Sachs patients compared to Sandhoff patients and were observed primarily in the cerebellum (FDR<0.05). No fiber tracts were evaluated to have a higher radial diffusivity in Sandhoff patients compared to Tay-Sachs patients (red).

medRxiv preprint doi: https://doi.org/10.1101/2024.12.13.24318793; this version posted December 16, 2024. The copyright holder for this preprint (which was not certified by peer review) is the author/funder, who has granted medRxiv a license to display the preprint in perpetuity. This article is a US Government work. It is not subject to copyright under 17 USC 105 and is also made available for use under a CC0 license.

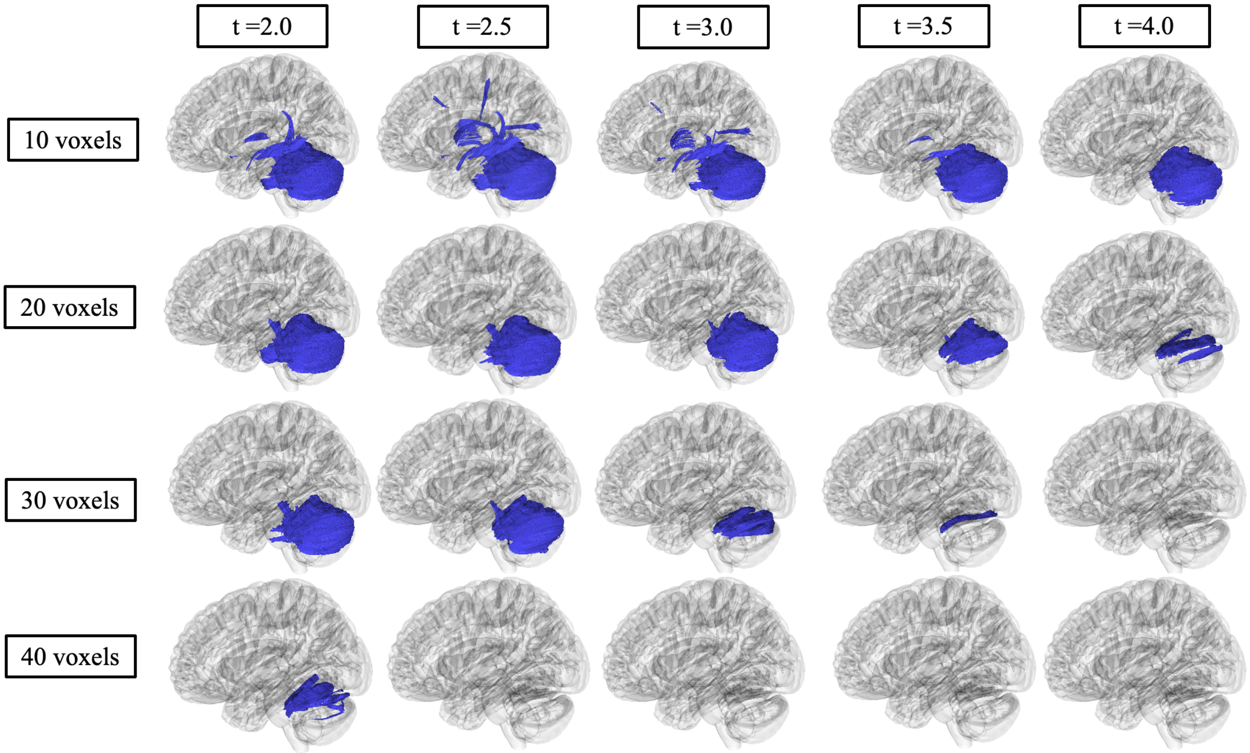

Figure D3. Correlational fiber tractography assessed differences in axial diffusivity (AD) in Sandhoff and Tay-Sachs patients at varying length (voxels) and T thresholds. Fiber tracts shown in blue were evaluated to have a higher axial diffusivity in Tay-Sachs patients compared to Sandhoff patients and were observed primarily in the cerebellum (FDR<0.05). No fiber tracts were evaluated to have a higher axial diffusivity in Sandhoff patients compared to Tay-Sachs patients (red).
